# Agrobacterium-Mediated Genetic Transformation of the Medicinal Plant *Veratrum dahuricum*

**DOI:** 10.3390/plants9020191

**Published:** 2020-02-04

**Authors:** Rui Ma, Zhijing Yu, Qinan Cai, Haiyun Li, Yingshan Dong, Kirsi-Marja Oksman-Caldentey, Heiko Rischer

**Affiliations:** 1 Jilin Provincial Key Laboratory of Agricultural Biotechnology, Jilin Academy of Agricultural Sciences, Changchun 130033, China; rui.ma@cjaas.com (R.M.); yuzhijing@cjaas.com (Z.Y.); caiqinan@cjaas.com (Q.C.); hyli@cjaas.com (H.L.); 2 VTT Technical Research Centre of Finland Ltd., P. O. Box 1000, FI-02044 VTT, Espoo, Finland; Kirsi-Marja.Oksman@vtt.fi

**Keywords:** *Veratrum dahuricum*, cyclopamine, *Agrobacterium tumefaciens*, transformation

## Abstract

*Veratrum dahuricum* L. (Liliaceae), a monocotyledonous species distributed throughout the Changbai mountains of Northeast China, is pharmaceutically important, due to the capacity to produce the anticancer drug cyclopamine. An efficient transformation system of *Veratrum dahuricum* mediated with *Agrobacterium tumefaciens* is presented. Murashige and Skoog (MS) medium containing 8 mg/L picloram was used to induce embryogenic calli from immature embryos with 56% efficiency. *A. tumefaciens* LBA4404 carrying the bar gene driven by the cauliflower mosaic virus 35S promoter was employed for embryogenic callus inoculation. *A. tumefaciens* cell density OD660 = 0.8 for inoculation, half an hour infection period, and three days of co-culture duration were found to be optimal for callus transformation. Phosphinothricin (PPT, 16 mg/L) was used as the selectable agent, and a transformation efficiency of 15% (transgenic plants/100 infected calli) was obtained. The transgenic nature of the regenerated plants was confirmed by PCR and Southern blot analysis, and expression of the bar gene was detected by RT-PCR and Quick PAT/bar strips. The steroid alkaloids cyclopamine, jervine, and veratramine were detected in transgenic plants, in non-transformed and control plants collected from natural sites. The transformation system constitutes a prerequisite for the production of the pharmaceutically important anticancer drug cyclopamine by metabolic engineering of *Veratrum*.

## 1. Introduction

*Veratrum dahuricum* (Liliaceae), often referred to as mountain corn, which was used as traditional Chinese medicine in the treatment of stroke, dysentery, jaundice, headache, and chronic malaria, grows in the Changbai mountains of Northeast China [1]. The genus *Veratrum* comprises up to 45 species of perennial herbs occurring throughout the northern temperate and Arctic regions of Europe, Asia, and North America [2]. A wide range of interesting pharmacological effects has been attributed to steroid alkaloids of *Veratrum* [1].

**Figure 1:**
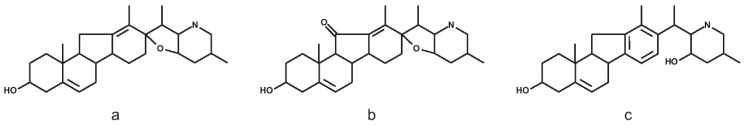
Chemical structures of cyclopamine (**a**), jervine (**b**), and veratramine (**c**).

Initially, veterinarians in the USA realized that a high percentage of sheep which had consumed *Veratrum californicum* during early pregnancy gave birth to deformed lambs, varying from cyclopia to mildly deformed upper jaws [3]. Cyclopamine (11-deoxojervine) and jervine (Figure 1a,b) turned out to be the responsible teratogens [4,5]. Dedicated investigation of cyclopamine-induced teratogens showed that the compound selectively blocks the sonic hedgehog (SHh) signaling pathway. This pathway is crucial in human embryogenesis and tissue differentiation [6]. Subsequently, cyclopamine and its derivatives have shown promising antineoplastic activities against several cancers that arise from the disruption of the SHh pathway [7], because several genes in the pathway have been associated with these tumors. Examples include pancreatic cancer [8,9], renal cell carcinoma [10], basal cell carcinoma [11,12], medulloblastoma tumor [13], small cell lung cancer [7], and leukemia [14]. Cyclopamine and its semi-synthetic analog, IPI-926, have entered clinical trials for the treatment of several cancers, including medulloblastoma, pancreatic cancer, and leukemia [9,14,15].

The cyclopamine supply is limited. The commercial cultivation of *V. californicum* has not been achieved so far and therefore wild populations are collected for extraction. Wild plants, however, contain highly variable quantities of the alkaloid. The chemical structure of cyclopamine is complex and chemical synthesis is not economic either. The biotechnological production of the compound is therefore an attractive alternative approach. Aided by the rapid development of metabolic engineering, it can be envisioned to produce the target compound by genetically engineered plants or corresponding cell, tissue, and organ cultures [16]. In vitro culture of *Veratrum* has already been established [17], and several functional genes covering the early steps in the biosynthesis of cyclopamine have been characterized [18]. However, a genetic transformation system of *Veratrum* has not been available, preventing the biotechnological improvement of compound production by genetic engineering so far.

In this paper we report a complete genetic transformation method for *V. dahuricum*, a species producing the pharmaceutically important steroid alkaloids cyclopamine, jervine, and veratramine (Figure 1a–c). The transformation system comprises embryogenic callus induction, callus infection by *Agrobacterium tumefaciens*, transgenic callus selection, and plant regeneration. The transformation method constitutes an indispensable tool for the functional analysis of genes involved in the steroidal alkaloid biosynthesis and the improved production of the anticancer drug cyclopamine by metabolic engineering.

## 2. Materials and Methods

### 2.1. Embryogenic Callus Induction

Immature seeds were harvested from *Veratrum dahuricum* (Turcz.) Loes.f growing in the Changbai Mountains of Northeast China at the end of July, and wild plants were collected in April. The seeds were surface sterilized with 70% (*v/v*) ethanol and then sterilized by 1.6% (*v/v*) sodium hypochlorite supplemented with a few drops of Tween20 for 15 min, followed by washing three times with sterile water. Immature embryos were excised under an anatomic microscope and cultured on modified Murashige and Skoog (MS) medium [19], consisting of MS basic salts and vitamins, 146 mg/L glutamine, 200 mg/L casein hydrolysate, solidified with 3 g/L phytagel, and supplemented with 8 mg/L picloram as growth regulator in the dark at 26 °C. Produced calli were subcultured to fresh plates at three to four weeks intervals. After three to four months of culture, the embryogenic calli were transferred to AA medium [20], supplemented with 4 mg/L of the phytohormone 2,4-D, solidified with 3 g/L phytagel, and subcultured to fresh plates at three to four weeks intervals.

### 2.2. Transformation and Plant Regeneration

**Figure 2:**
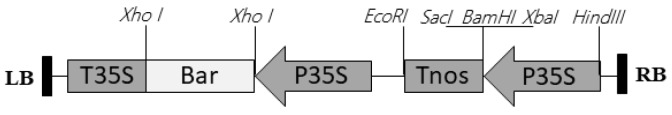
T-DNA region of the expression vector pCAMBIA3300-35S-bar, containing a selectable marker gene *bar* (phosphinothricin acetyl transferase) driven by the cauliflower mosaic virus 35S promoter (P35S).

The fast growing and fresh yellow embryogenic calli (subcultured for about 3 weeks) were used as explants for infection. *Agrobacterium tumefaciens* strain LBA4404 harboring a binary vector pCAMBIA3300 (Figure 2) was employed for the transformation. The vector pCAMBIA3300 contains a bar gene expression cassette which confers resistance to the herbicide phosphinothricin (PPT). The *Agrobacterium* was grown on LB solid medium containing 100 mg/L kanamycin, 50 mg/L rifampicin and 50 mg/L streptomycin for 2–3 days at 28 °C. The bacterial cultures were harvested and suspended in the modified MS liquid medium with 0-100 µM acetosyringone. The *Agrobacterium* suspensions were adjusted to different cell densities (OD600 = 0.2, 0.3, 0.5. 0.8, 1.0, 1.2, 1.4).

The embryogenic calli in a Petri dish were immersed in 20 mL of the bacterial suspension and incubated at 24 °C on a rotary shaker (30 rpm) for 30 min. The inoculated calli were blotted dry on sterile filter paper, then transferred to co-culture medium (modified MS medium with 0–100 µM acetosyringone, 30 g/L sucrose and 3 g/L phytagel and a sterilized filter paper). After co-culture for 3 days in dark at 25 °C, the co-cultured calli were washed with sterile water, blotted dry and then transferred to selection medium (AA medium with 4 mg/L 2,4-D, 30 g/L sucrose, 3 g/L phytagel, 250 mg/L ticarcillin, 100 mg/L cefotaxime and 16 mg/L PPT). The cultures were kept in the dark, at 25 °C and were subcultured at 4 week intervals.

After 3–4 cycles of selection, the PPT resistant calli were transferred to solid regeneration medium R2M [21] with 3 mg/L 6-BA, 3 g/L phytagel, 8 mg/L phosphinothricin, and cultured in light (52 µmol m^−2^ s^−1^; cool white) at 25 °C. After 4–5 weeks, green shoots were moved to hormone-free medium R2M in a 250 mL Erlenmeyer flask for root development. Finally, after another 1 month, well-developed plantlets were individually potted into peat soil and transferred to the greenhouse.

### 2.3. PCR Analysis

Total genomic DNA was isolated from leaves of the putative transgenic plantlets and non-transformed plantlets using the Plant Genomic DNA Kit (Tiangen Biotech, Beijing, China), according to the supplier extraction protocol. *E. coli* and *Agrobacterium tumefaciens* LBA4404 plasmid DNA was isolated using Qiagen Plasmid mini Kit following the instructions. The specific primers used for bar and virD gene amplification are listed in Supplementary Table S1. Expected sizes of the amplified fragments were 441 bp and 477 bp, respectively. The PCR reaction mixture (25 µL) contained 50 ng of genomic DNA or 10 pg plasmids, 0.5 µL of 10 mM dNTP, 2.5 µL of 10× PCR buffer, 1 µL each of 10 mM forward and reverse primers, 0.2 µL of 5 U/µL Taq enzyme (Tiangen Biotech, Beijing, China) and 18.8 µL ddH2O. The PCR condition for bar gene amplification was 94 °C for 5 min, 94 for 45 s, 55 °C for 45 s, 72 °C for 1 min with 35 cycles, following 72 °C for 10 min. The PCR condition for virD gene amplification was 94 °C for 5 min, 94 for 45 s, 58 °C for 1 min, 72 °C for 1 min with 35 cycles, following 72 °C for 10 min.

### 2.4. Reverse Transcription-PCR (RT-PCR) Analysis

Total RNA was isolated from leaves of the transgenic plantlets and the control non-transformed plantlets following the protocol of Plant RNA Extract Kit (Invitrogen) and then treated with amplification grade DNaseI (Invitrogen) to remove all DNA. RNA was reverse transcribed to cDNA with a TransScript one-step gDNA Removal and cDNA synthesis SuperMix Kit (TransGen, Beijing, China).

The first-strand cDNA was then diluted tenfold to amplify a specific 441 bp bar fragment following the protocol mentioned before for genomic DNA amplification using the same primer pair.

### 2.5. Southern Blot Analysis

Total genomic DNA of the putative transgenic plantlets and non-transformed plantlets was extracted using a modified CTAB (cetyltrimethylammonium bromide) method [22]. Genomic DNA (30 µg) was digested with EcoR I, then subjected to electrophoresis in 0.8% (*w*/*v*) agarose gels and subsequently transferred to positively charged nylon membranes (GE Amersham, Beijing, China) by capillary transfer following the manufacturer’s instructions. The expression vector and non-transformed plantlet were used as positive and negative controls respectively. A DIG-labelled bar probe (the PCR product with 441 bp amplified from plasmid as above) was produced by PCR labelling. Subsequent hybridization steps were carried out with the DIG High Prime DNA Labelling and Detection Starter Kit II (Roche, Germany). Hybridization was carried out at 42 °C for 16 h and staining was performed at room temperature with NBT/BCIP as substrate (following the instruction of the DIG High Prime DNA Labelling and Detection Starter Kit I, NBT /BCIP chromogenic method).

### 2.6. Bar Protein Detection

In order to detect the bar protein expression quickly, fresh leaves of the putative transgenic and non-transformed plantlets were excised in a 1.5 mL Eppendorf tube. The bar protein was detected by using the Quick Stick Kit for LibertyLink (bar) (ENVIROLOGIX, Portland, Maine, USA) according to the protocol of the manufacturer.

### 2.7. Extraction and Targeted HPLC Analysis

Of the lyophilized plant material, 100 mg were milled into powder and suspended in 4 mL ethanol (80%, at ratio 1:4 *w*/*w*) for 1 h, 16 mL ethanol (95%, at ratio 1:4 *w*/*w*) was added, and the sample was extracted by agitation reflux for 2 h at 80 °C. Following filtration, the filtrate was collected and the sample residue was re-extracted twice with another 16 mL ethanol (95%, at ratio 1:8 *w/w*). The filtrates were combined and the solvent was evaporated to dryness. The extract was re-dissolved in 200 mL of methanol in an ultrasonic bath (Power 250 W, Frequency 40 kHz) for 1 min, and then filtered using a 0.22 µm membrane. The filtrate was collected for HPLC analysis.

HPLC separation was performed using an Agilent 1260 Infinity system with a VWD monitor. An aliquot of 20 µL of sample was loaded onto a reverse-phase C18 column (Diamonsil C18, 4.6 × 250 mm, 5 µm stainless steel, Diamonsil Science and Technology Company, Beijing, China) at 30 °C. The sample was eluted within 30 min using isocratic conditions of methanol and 0.1 M ammonium acetate (60:40, *v*/*v*) applying a flow of 1 mL/min. UV detection was operated at a wave length of 220 nm. Commercially available alkaloids cyclopamine, jervine, and veratramine (LC Laboratories, Woburn, Maryland, USA) were used as reference compounds.

### 2.8. Statistical Analysis

Frequencies of immature embryos producing embryogenic calli (embryogenic calli/100 explants), frequencies of growing embryogenic calli in the experiment for PPT selection effects, and frequencies of transformation (transgenic plants/100 infected calli) in the experiments for influencing parameters optimization were recorded. Completely randomized designs (CRD) were used in the experiments. Each treatment contained five replicates. Data analyses with two treatment levels were carried out by t-test and with more than two treatment levels by the ANOVA procedure. Multi-range comparisons were performed by Fisher’s Least Significant Difference (LSD) test.

## 3. Results and Discussion

### 3.1. Embryogenic Callus Induction

In embryo cultures of monocotyledonous plants, induction of somatic embryogenesis is generally influenced by several factors including, e.g., embryo size [23,24], culture medium [25,26,27], and growth regulators [24,25,28]. For optimal results, these parameters must be empirically determined. Considering *Veratrum*, only conditions for *V. californicum* had been published [17]. In this case, embryos with 2-4 mm size cultured on modified MS medium with picloram showed the best results for embryogenic callus production [17].

**Figure 3:**
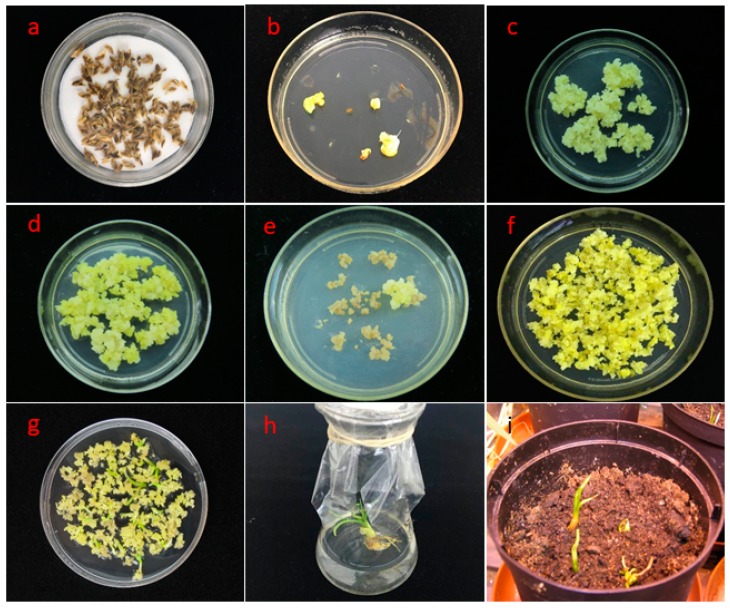
Genetic transformation of *Veratrum dahuricum* mediated with *Agrobacterium tumefaciens* carrying pCAMBIA3300-35S-bar expression vector. (**a**) Surface sterilized seeds of *Veratrum dahuricum*; (**b**) embryogenic calli induction on modified MS-medium containing 8 mg/L picloram; (**c**,**d**) proliferation of embryogenic calli in AA medium containing 4 mg/L picloram; (**e**) transgenic callus selection on AA medium containing 4 mg/L 2,4-D and 16 mg/L PPT; (**f**) growing transgenic calli with bar gene on AA selection medium; (**g**) green plant regeneration on R2M medium containing 3 mg/L 6-BA; (**h**) rooted plantlets on hormone free medium R2M; (**i**) potted plantlets in peat soil.

We expected that similar conditions could work for *V. dahuricum* and, indeed, embryogenic calli were successfully induced from isolated immature embryos (embryo size 2–3 mm) of surface sterilized seeds (Figure 3a) on modified MS medium with 8 mg/L picloram (Figure 3b). The induction frequency of embryogenic calli (embryogenic calli/100 explants) was 56% (n = 520). The embryogenic calli with a high regeneration potential were successfully proliferated by subculturing at 3–4 week intervals (Figure 3c,d). The efficiency for green plant regeneration (green plantlets/100 calli) was 95% (n = 254).

### 3.2. Determination of PPT Concentration for Transgenic Callus Selection

PPT, together with the bar gene which confers resistance to PPT, is widely used as a selection agent in plant genetic transformation for example in crops such as maize, rice, wheat, and soybean [29,30,31,32]. Typically, concentrations in selection media vary from 2–6 ppm.

**Figure 4:**
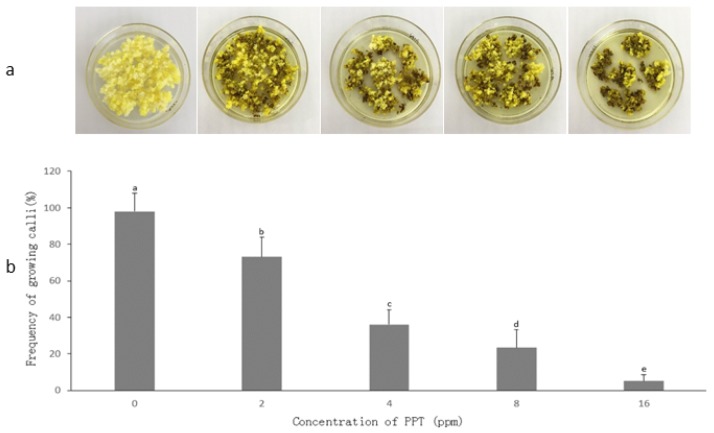
Selection effect of PPT concentrations on calli of *Veratrum dahuricum* after 4-week culture. (**a**) Calli on selection medium with different PPT concentrations, (**b**) selection efficiency of different PPT concentrations (n = 690). Treatments (means ± SD) followed by a different letter are significantly different according to the LSD test (*p* < 0.05).

Initial experiments were conducted to establish the suitable PPT concentration in the selection medium of *V. dahuricum* to select putative transgenic calli. Interestingly, the embryogenic calli of *V. dahuricum* displayed low sensitivity to PPT compared to the plants mentioned above. A suitable PPT concentration was 16 mg/L resulting in almost complete inhibition of callus growth within four weeks (about 5.1% surviving calli, n = 690, Figure 4). Hence, the concentration of 16 mg/L PPT in selection medium was used in subsequent transgenic callus selection.

### 3.3. Optimization of Agrobacterium Cell Density

Cell density of *Agrobacterium* is another important factor influencing genetic transformation. In previous studies for plant transformation, such as in rice [30], maize [33], soybean [32], wheat [34], clove basil [35], and jute [36], optimum cell densities varied from OD = 0.1–OD = 1.0 depending on *Agrobacterium* strains, plant species, and explants.

**Figure 5:**
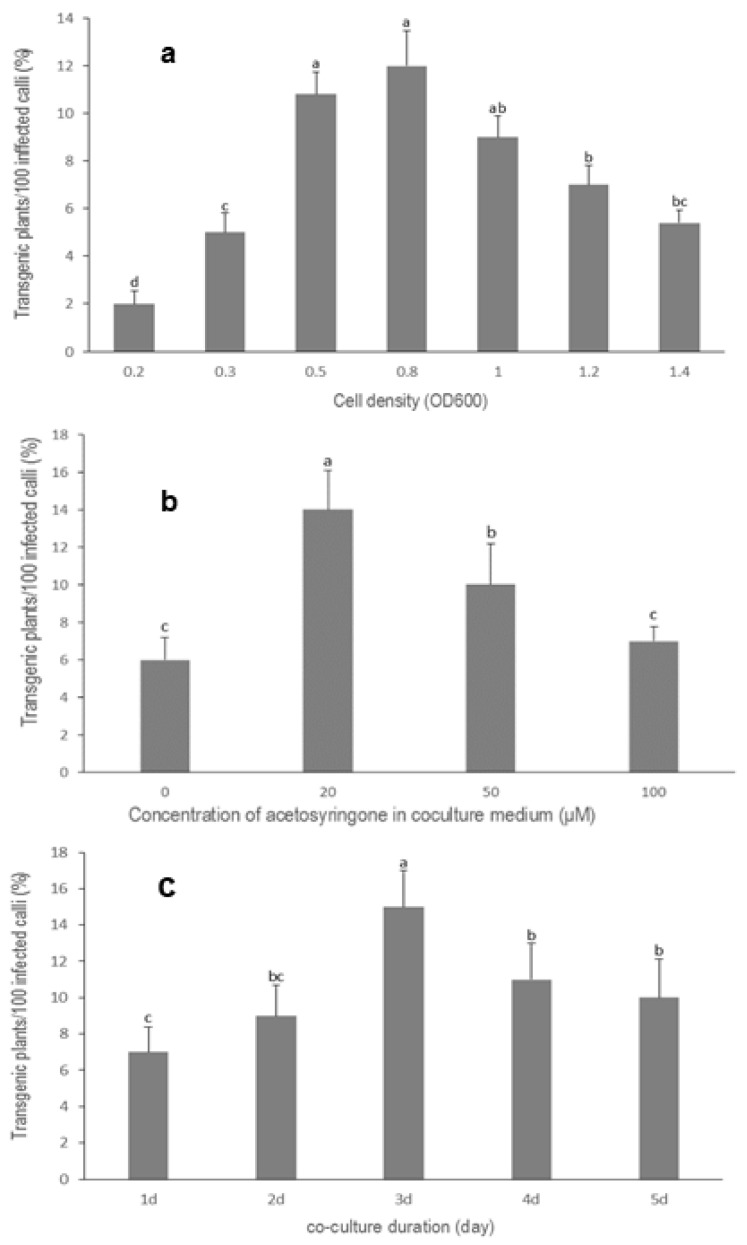
Factors influencing transformation efficiencies of *Veratrum dahuricum*. Treatments (means ± SD) followed by a different letter are significantly different according to the LSD test (*p* < 0.05). (**a**) Influence of *Agrobacterium* cell density on transformation efficiency (n = 933), (**b**) effects of acetosyringone on transformation efficiency (n = 500), (**c**) influence of co-culture duration on transformation efficiency (n = 633).

For *V. dahuricum* an *Agrobacterium* density of OD600 < 0.5 was ineffective for the transformation, whereas OD600 > 1.0 led to the death of the calli, due to overgrowth of bacteria. The highest transformation frequency of 12% (n = 933) was achieved with a cell density of about OD600 = 0.8 (Figure 5a).

### 3.4. Effect of Acetosyringone on Transformation Efficiency

Acetosyringone, a phenolic compound exuded from plant wounds, has been used in *Agrobacterium*-mediated transformation during infection to increase transformation frequency [33,37,38]. Acetosyringone concentrations typically range from 20–200 µM, depending on the plant species [32,33,37,38].

In the case of *V. dahuricum*, the transformation efficiency was significantly increased by the addition of acetosyringone to the infection and co-culture medium. Different concentrations of acetosyringone in the range of 0–100 µM were added to the co-culture medium at the infection time. The highest transformation efficiency (14%, n = 500) was achieved at a concentration of 20 µM acetosyringone (Figure 5b).

### 3.5. Influence of Co-Culture Duration on Transformation Efficiency

In the *Agrobacterium*-mediated genetic transformation of plants, transfer of T-DNA from *Agrobacterium* to the plant is a time-dependent process and the duration varies widely from a few hours to a few days, depending on the *Agrobacterium* strain and the plant species [39]. Co-culture duration is therefore one of the main factors affecting transformation efficacy. In many plant transformation studies, e.g., in maize [40], rice [30], wheat [34], soybean [41], okra [39], and clove basil [35], suitable co-culture duration varied from two to five days.

In our studies, co-culture duration significantly influenced the transformation efficiency of *V. dahuricum*. Within the period of five days, there was a clear optimum at day three (Figure 5c). Approximately 15% transgenic plants were recovered (n = 633). A further increase of co-culture duration led to lower transformation frequency, due to visible overgrowth of *Agrobacterium*.

### 3.6. Transgenic Callus Selection and Transformed Plant Regeneration

Transgenic callus selection and maintenance of embryogenic capacity and regeneration potential have been a critical problem to overcome in the establishment of efficient transformation systems [23].

In our study with *V. dahuricum*, infected calli were moved to selection medium AA after co-culture. Following three to four cycles of selection, PPT resistant calli were obtained (Figure 3e,f) and plated on solid regeneration medium R2M. Already after another four to five weeks, green shoots regenerated and these were transferred to hormone-free R2M medium in 250 mL Erlenmeyer flasks for root development (Figure 3g). Fully developed plantlets (Figure 3h) were then potted in peat soil and successfully weaned in the greenhouse (Figure 3i).

### 3.7. Molecular Analysis of Putative Transgenic Plants

**Figure 6:**
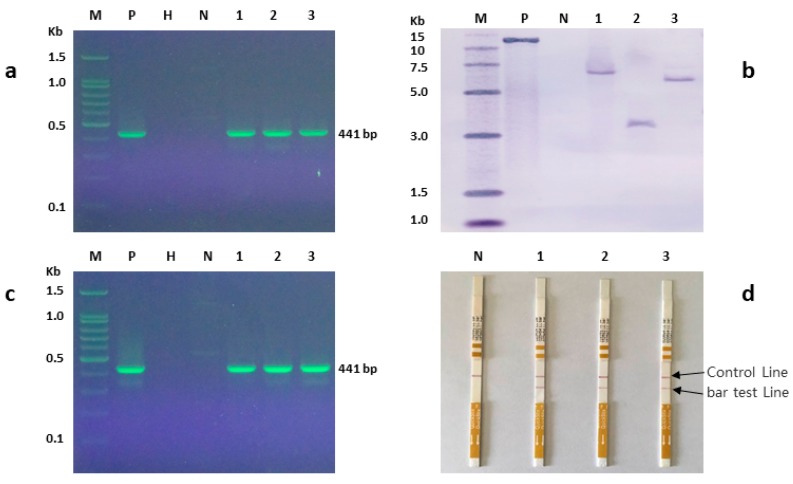
Molecular analysis of *Veratrum dahuricum* regarding *bar* (phosphinothricin acetyl transferase) gene. M: DNA molecular weight marker, P: positive control of plasmid pCAMBIA3300-35S-bar, H: negative water control, N: non-transformed control plant, 1–3: transgenic plants (**a**) PCR analysis showing the expected 441 bp band in the positive control and transgenic plants 1–3; (**b**) Southern blot analysis showing the single hybridized band in the positive control and transgenic plants 1–3. Plant genomic DNA and plasmid were digested with *EcoR I* and hybridized with digoxigenin (DIG) labelled *bar* probe. (**c**) Expression analysis of *bar* gene in transgenic plants by RT-PCR using bar gene primers. The expected 441 bp band is visible in the positive control and transgenic plants 1–3; (**d**) Bar protein expression analysis by ENVIROLOGIX quick stick kit for LibertyLink (bar). Transgenic plants 1–3 showing BAR.

Putative transformed plants were evaluated by PCR and Southern blot. The PCR amplified DNA fragment of the bar gene from transgenic plants was 441 bp which was in accordance with the expected amplification size. DNA from a non-transformed control plant did not show any amplified fragment (Figure 6a). The transgenic nature of the plants was further confirmed by Southern blot hybridization. DNA from transgenic plants showed hybridization fragments with one (Figure 6b) to three copies (Supplementary Figure S2), whereas DNA from non-transformed control plants did not generate any hybridization band (Figure 6b). These results indicated that the bar gene was successfully integrated into the genome of *V. dahuricum*. Three selected transgenic plants with a single copy of the bar gene showed the expected 441 bp fragment amplified by qualitative RT-PCR which demonstrated that the bar gene was indeed transcribed (Figure 6c). The Bar protein was expressed in the transgenic *V. dahuricum* plants, while a non-transformed control plant did not show Bar protein test line (Figure 6d). Plants were free of persisting *Agrobacterium*, as shown by virD PCR (Supplementary Figure S3).

### 3.8. Cyclopamine Content of Transgenic Plants

The steroid alkaloids cyclopamine, jervine, and veratramine were extracted from transgenic plants, non-transformed control plants, and wild collected plants. The contents of the alkaloids in the plants were determined by targeted HPLC-UV. All three alkaloids were detected in the examined plants but significant quantitative differences (*p* > 0.05) were not found (Table 1, Supplementary Figure S4).

All three alkaloids, cyclopamine, jervine, and veratramine have earlier been found in *V. californicum*. Biosynthetically cyclopamine derives from cholesterol, while both veratramine and jervine are subsequent derivatives of cyclopamine [42]. The contents of cyclopamine in wild collected *Veratrum* plants vary considerably from 39–800 mg/100 g dw depending on the location and growth stage [43,44]. In the present study contents of cyclopamine in transgenic and non-transformed and wild collected control plants were rather low: 3.89, 3.83, and 4.24 mg/100 g dw, respectively. Although with a low likelihood, T-DNA insertional mutagenesis can knock out biosynthetic genes [45] and even adjacent integration may affect expression levels [46]. The transformation of *V. dahuricum* with only a selection marker did not alter cyclopamine and total alkaloid contents and therefore an influence of the integrated gene on biosynthetic genes is not apparent.

## 4. Conclusions

The establishment of transformation protocols is still a largely empiric, yet indispensable exercise in order to be able to metabolically engineer non-model plant species capable of producing interesting secondary metabolites. Among the monocotyledonous *Veratrum* species, *V. californicum* and *V. dahuricum* contain pharmaceutically important steroid alkaloids. While basic plant regeneration methods exist for *V. californicum*, this study presents for the first time an efficient *Agrobacterium*-mediated genetic transformation system for *Veratrum*. A number of relevant factors, such as the size of explant, concentration of the selection marker, *Agrobacterium* cell density, acetosyringone concentration, and co-culture duration were optimized to achieve regenerated transgenic plants that were subsequently successfully weaned in the greenhouse. Transgenic and control plants contained similar amounts of the target molecules, confirming that transformation with a marker gene does not disrupt the capacity to produce these compounds. Further work will focus on the introduction of biosynthetic pathway genes that aim at modulating steroid alkaloid accumulation.

## Figures and Tables

**Table 1:** Contents of steroid alkaloids in different plant sources.

Plant Source	Contents of Steroid Alkaloids (mg/100 g dw)			
	Cyclopamine	Jervine	Veratramine	Total
Transgenic plant	3.89a	12.79a	26.54a	43.22a
Non-transformed control plant	3.83a	13.56a	25.04a	42.43a
Wild collected control plant	4.24a	11.43a	23.36a	39.03a

Means (n = 6) followed by the same letters are not significantly different according to the LSD test (*p* < 0.05).

## Supplementary Materials

The following is available online at https://www.mdpi.com/2223-7747/9/2/191/s1, Table S1: Primers for PCR and RT-PCR; Figure S2: Southern blot analysis showing 1–3 hybridized bands; Figure S3: PCR detection of virD; Figure S4: HPLC analysis of steroid alkaloids cyclopamine, jervine, and veratramine in the transgenic plant, non-transformed control plant, and wild collected plant of *Veratrum dahuricum* at wavelength 220 nm.
