# Supplementary material for: Agrobacterium-Mediated Genetic Transformation of the Medicinal Plant Veratrum dahuricum
Source: Plants (Basel). 2020 Feb 4;9(2):191. doi: 10.3390/plants9020191 (PMC7076492; doi:10.3390/plants9020191)
Supplement: Supplementary file 1 [file plants-09-00191-s001.pdf]

plants

Supplementary Material:
Rui Ma et al. Agrobacterium-mediated Genetic Transformation of the Medicinal Plant *Veratrum dahuricum*

Supplementary Table S1. Primers for PCR and RT-PCR.

Gene	Primers	Expected Size
bar (for PCR and RT-PCR)	BarF: 5′-GCACCATCGTCAACCACTACATCGAG-3′<br>BarR: 5′-TGAAGTCCAGCTGCCAGAAACCCAC-3′	441 bp
VirD	VirDF: 5′-TAGCAGAGCGACCAATCACA-3′<br>VirDR: 5′-GACCTGCCTCCTCGTCATTA-3′	477 bp

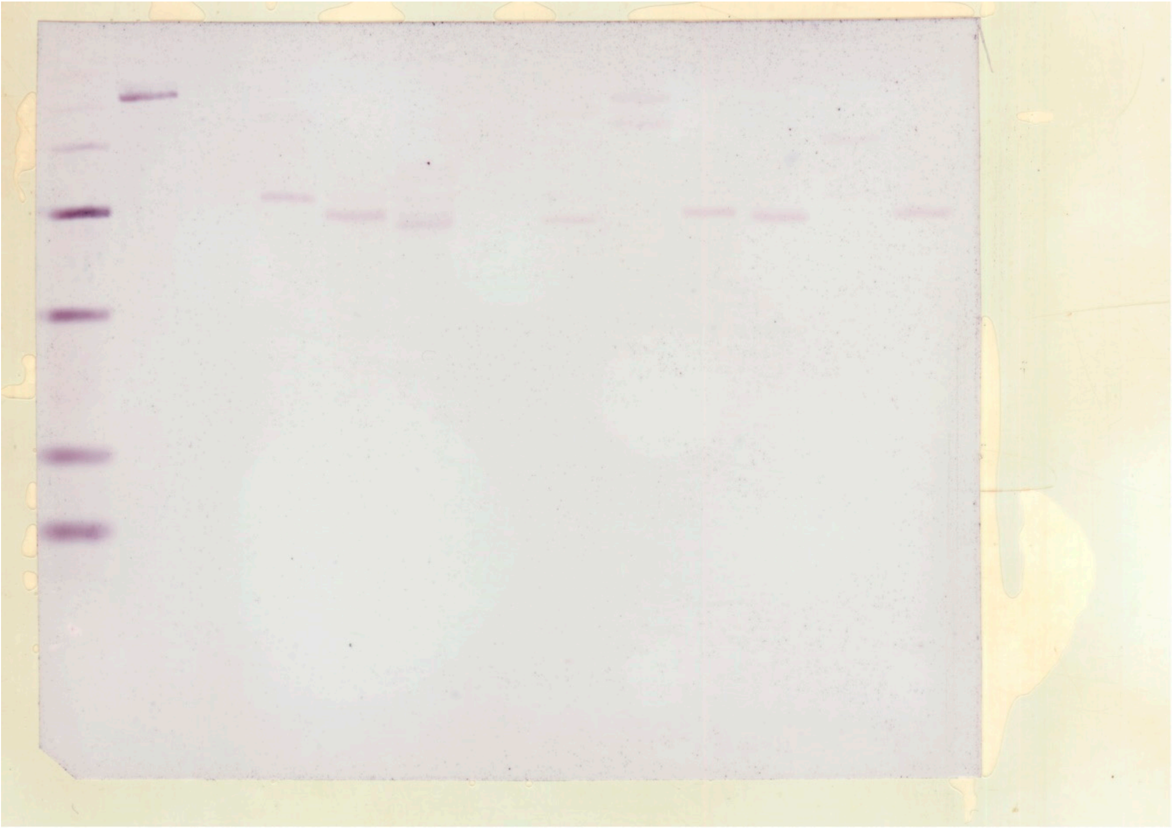

Supplementary Figure S2. Southern blot analysis showing 1-3 hybridized bands.

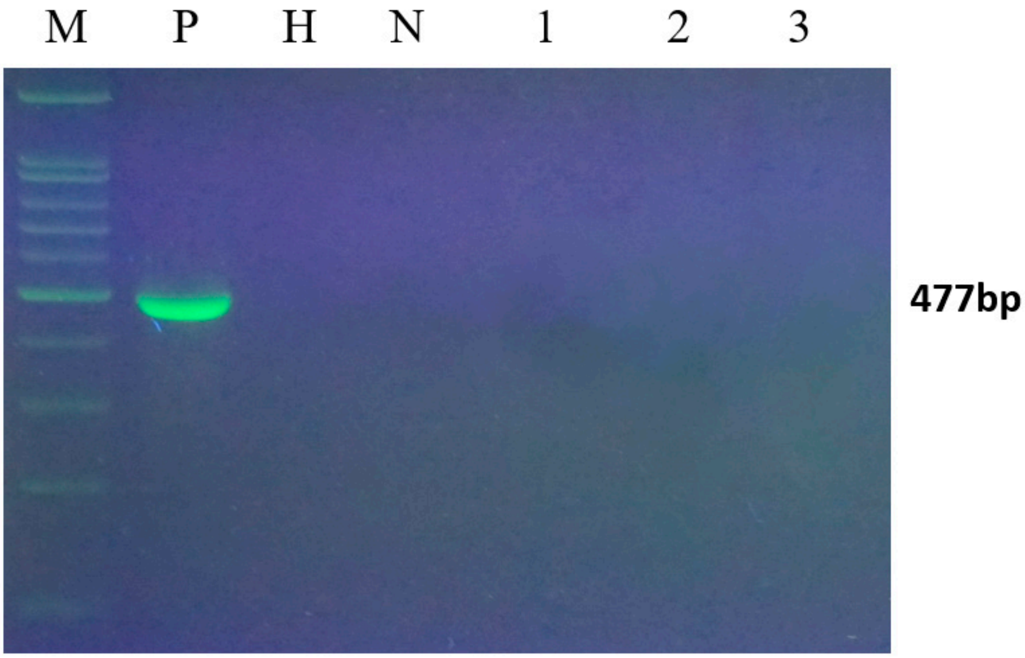

Supplementary Figure S3. PCR detection of *virD* gene of *Agrobacterium* strain LBA4404, showing the expected 477bp band in the positive control and no bands for negative water control, non-transformed control plant and transgenic plants. M: DNA molecular weight marker, P: positive control of plasmid pAL4404, H: negative water control, N: non-transformed control plant, 1- 3: transgenic plants. PCR analysis.

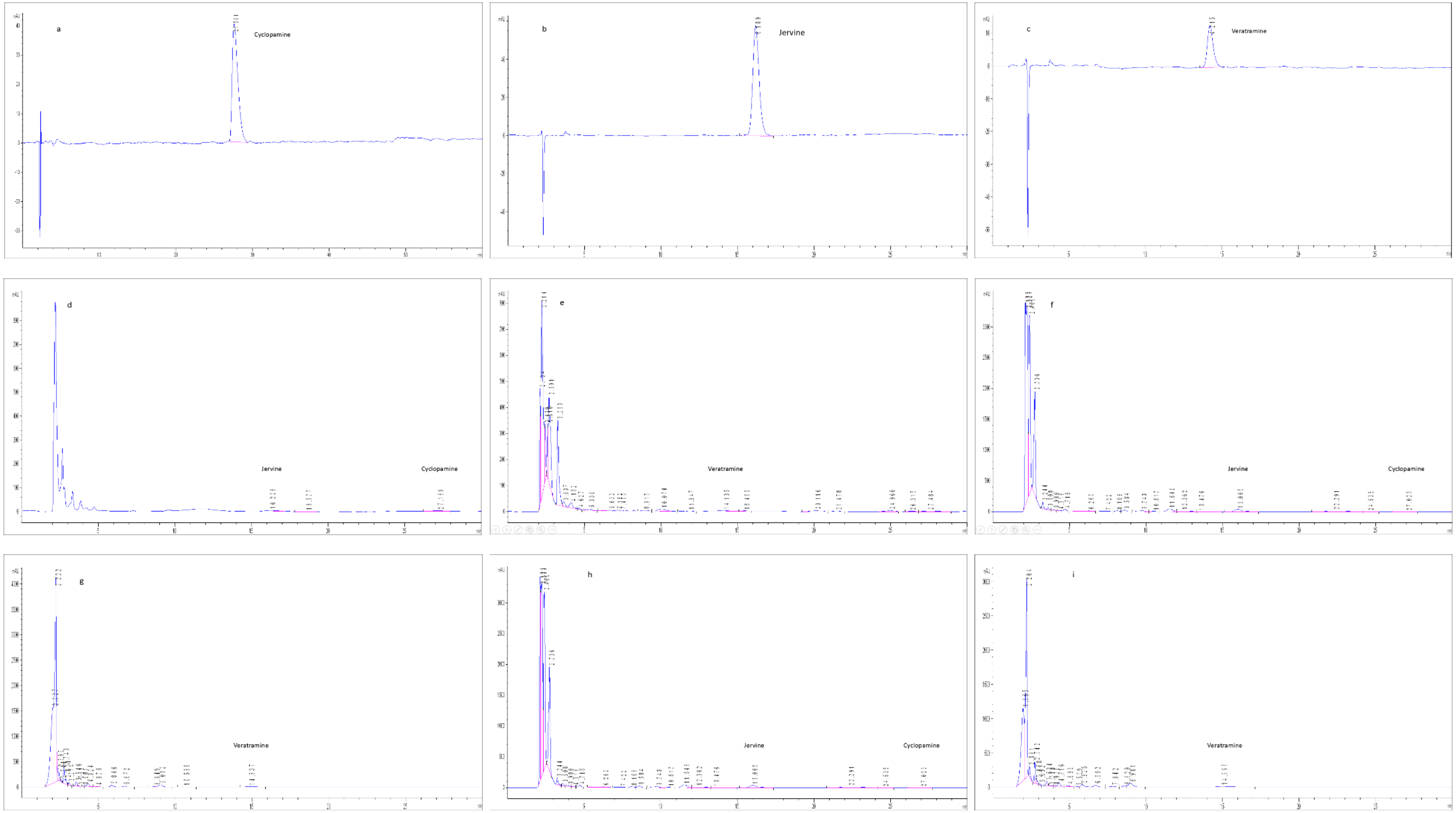
a
Cyclopamine
b
Jervine
c
Veratramine
d
Jervine
Cyclopamine
e
Veratramine
f
Jervine
Cyclopamine
g
Veratramine
h
Jervine
Cyclopamine
i
Veratramine

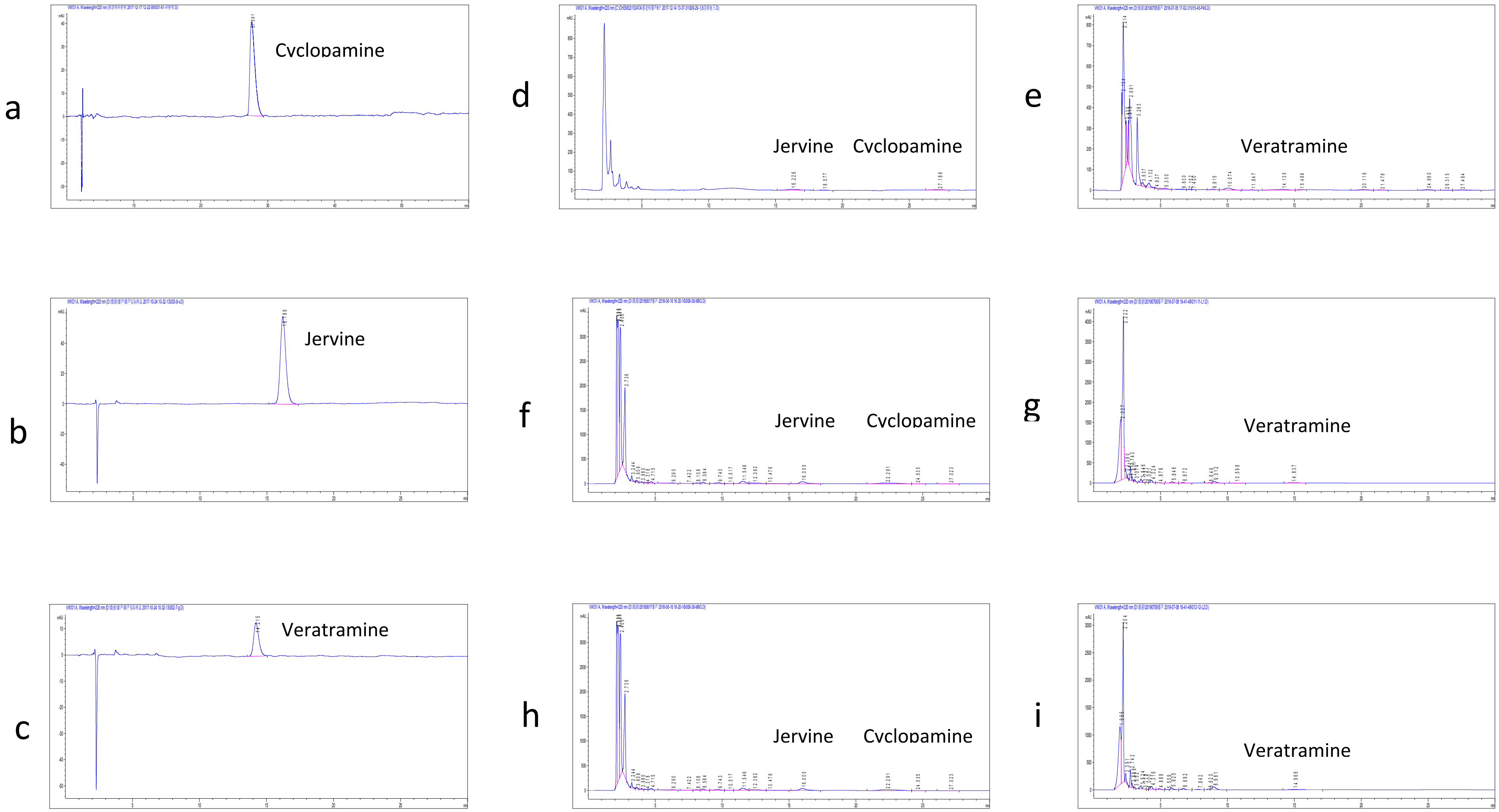
a
Cyclopamine
b
Jervine
c
Veratramine
d
Jervine
Cyclopamine
f
Jervine
Cyclopamine
h
Jervine
Cyclopamine
e
Veratramine
g
Veratramine
i
Veratramine

Supplementary Figure S4. HPLC analysis of steroid alkaloids cyclopamine, jervine and veratramine in transgenic plant, non-transformed control plant and wild collected plant of *Veratrum dahuricum* at wavelength 220 nm. (**a**), (**b**), (**c**) Chromatograms of commercial reference alkaloids cyclopamine, jervine and veratramine; (**d**), (**e**) Chromatograms of crude alkaloid extracts derived from transgenic plants (transgenic plant 1 and 2). Jervine, cyclopamine and veratramine were detected at retention time 16.22, 27.18 and 14.13min, respectively; (**f**), (**g**) Chromatograms of crude alkaloid extracts derived from non-transformed control plant. Jervine, cyclopamine and veratramine were detected at retention time 16.05, 27.02 and 14.83min, respectively; (**h**), (**i**) Chromatograms of crude alkaloid extracts derived from wild collected plant. Jervine, cyclopamine and veratramine were detected at retention time 16.00, 27.02 and 14.98min, respectively.
